# Emergence, development and diversification of the TGF-*β *signalling pathway within the animal kingdom

**DOI:** 10.1186/1471-2148-9-28

**Published:** 2009-02-03

**Authors:** Lukasz Huminiecki, Leon Goldovsky, Shiri Freilich, Aristidis Moustakas, Christos Ouzounis, Carl-Henrik Heldin

**Affiliations:** 1 Ludwig Institute for Cancer Research, Uppsala University, Uppsala, Sweden; 2 Computational Genomics, Institute of Agrobiotechnology, Thessaloniki, Greece

## Abstract

**Background:**

The question of how genomic processes, such as gene duplication, give rise to co-ordinated organismal properties, such as emergence of new body plans, organs and lifestyles, is of importance in developmental and evolutionary biology. Herein, we focus on the diversification of the transforming growth factor-*β *(TGF-*β*) pathway – one of the fundamental and versatile metazoan signal transduction engines.

**Results:**

After an investigation of 33 genomes, we show that the emergence of the TGF-*β *pathway coincided with appearance of the first known animal species. The primordial pathway repertoire consisted of four Smads and four receptors, similar to those observed in the extant genome of the early diverging tablet animal (*Trichoplax adhaerens*). We subsequently retrace duplications in ancestral genomes on the lineage leading to humans, as well as lineage-specific duplications, such as those which gave rise to novel Smads and receptors in teleost fishes. We conclude that the diversification of the TGF-*β *pathway can be parsimoniously explained according to the 2R model, with additional rounds of duplications in teleost fishes. Finally, we investigate duplications followed by accelerated evolution which gave rise to an atypical TGF-*β *pathway in free-living bacterial feeding nematodes of the genus Rhabditis.

**Conclusion:**

Our results challenge the view of well-conserved developmental pathways. The TGF-*β *signal transduction engine has expanded through gene duplication, continually adopting new functions, as animals grew in anatomical complexity, colonized new environments, and developed an active immune system.

## Background

Most genes belong to gene families, which have emerged through consecutive cycles of gene duplications during evolution [1]. With the availability of entire genome sequences, much progress has been made towards the understanding of gene duplication dynamics [2,3] and the evolutionary forces responsible for the retention of a proportion of duplicate genes, such as neo-functionalization [4] and sub-functionalization [5], both at the level of gene expression patterns [6,7] and protein sequence evolution [8]. However, further investigation is required to understand how genomic processes, such as gene duplications and losses, result in higher-level co-ordinated molecular events, such as the emergence of novel signal transduction pathways, which in turn give rise to phenotypic innovations, such as novel organs, developmental phases, or body plans.

To approach this question from a comparative genomics viewpoint, we focus herein on the emergence and evolution of the transforming growth factor-*β *(TGF-*β*) pathway within the animal kingdom. This pathway has been recognized as one of the fundamental and versatile metazoan signal transduction engines, with central roles in development, organogenesis, stem-cell control, immunity, and cancer [9-11]. A concise description of the human pathway has been deposited by the authors of this article in the Reactome [12] knowledge base [13].

The cellular core of all TGF-*β *superfamily pathways consists of cell surface receptors, called type I and type II serine-threonine kinase receptors, and intracellular Smad proteins [14]. The latter constitute the actual signal transduction engine of the pathway [15]. There are eight known Smads in the human genome, classified as: two TGF-*β sensu stricto *(Smad2,3) and three bone morphogenetic protein (BMP)-type (Smad1,5,8) receptor-activated Smads (R-Smads), one common mediator Smad (Co-Smad; Smad4), and two inhibitory Smads (I-Smads; Smad6,7). These eight genes are highly similar in sequence and are evidently results of multiple gene duplications of unknown origins. While the functional differences between the three biochemical classes of Smads are well known, their evolutionary history, the characteristics of the ancestral Smad molecule, and the selection forces behind the retention of multiple subtypes of R- and I-Smads are poorly understood.

In humans, we encounter five distinct type II receptors and seven distinct type I receptors [16]. The functional receptor unit is a hetero-tetramer of two type II receptors with two type I receptors, in which upon binding of the ligand the type II receptors phosphorylate the type I receptors, while the latter phosphorylate and activate R-Smads. Analysis of the receptor genes, so far has been limited to a few species, namely humans, rodents, African clawed frog (*Xenopus laevis*), fruit fly (*Drosophila melanogaster*) and the free-living roundworm *Caenorhabditis elegans *[17].

Mammalian genomes encode up to 33 TGF-*β *related ligands, *D. Melanogaster *seven and *C. elegans *five (out of which only two are functionally characterised) [18]. However, we do not focus on TGF-*β *related proteins in this study, as these sequences are rather diverged (and similarity is mostly confined to the carboxyterminal polypeptide of the much larger precursor proteins) rendering them difficult to analyse in multiple genomes using a unified computational pipeline. We refer the interested reader to a review by Herpin et al [19]. The most prevalent mode of extracellular modulation of TGF-*β *signalling is by means of soluble antagonists, called ligand traps, such of those of the chordin and gremlin family [20]. BAMBI is another important negative regulator of TGF-*β *signalling, related to TGF-*β *family type I receptors but lacking an intracellular kinase domain [21].

BMP signalling gradients, modulated by chordin, have been found to induce dorsoventral axis formation in the Spemann organizer [22]. Thus, traditionally, the TGF-*β *pathway had been thought to have evolved in the context of dorsoventral patterning, and thus be present only in Bilateria. This view has been recently challenged by the discovery of the functional pathway in multiple cnidarians [23-29]. Furthermore, the origin of animals themselves is only now being understood (for reviews see [30,31]). On the basis of mitochondrial DNA sequence comparison, the choanoflagellates have been identified as the closest single-celled animal relatives [32,33] while the Placozoan *Trichoplax adhaerens*, the so-called tablet animal [34,35], has been placed at the root of animal phylogeny [34,36]. However, some authors regard sponges as earlier diverging than Placozoans [37,38]. Regardless of the relative position of Placozoans and sponges, the critical step of transformation to multicellulararity must have been accompanied by the development of adhesion molecules, extracellular matrix proteins (such as collagen), and cell-to-cell communication. It is essential to identify the critical signalling pathways, in particular those involved in control of development, cellular differentiation and body plan formation [31]. Such comparisons will not only shed light on metazoan origins, and advance the field of evo-devo, but will also help us understand the fundamental functional motifs that underlie interwoven signal transduction networks of higher animals, with impact on human health.

It was reported previously that atypical dauer pathway Smads could be found in free-living bacterial feeding nematodes of the genus Rhabditis (Rhabditoid nematodes) [39]. The dauer (German for resting) is a survival and dissemination form, formed by all Rhabditoid nematodes, an alternative to the active third stage larvae (L3). Dauers are induced by environmental stress factors, such as lack of food, overcrowding, or high temperature. The dauer pathway (which also includes insulin pathway-like and guanyl cyclase pathway-like genes) is of high general interest, as it has been linked with aging [40], biodiversity [41] and the development of parasitism in nematodes [42]. However, the origins of the dauer pathway Smads had been previously unknown.

## Results

### TGF-*β *pathway gene content across taxa

Using the full genome sequences of 33 species (Table 1), we performed a comparative analysis of the TGF-*β *pathway genes, focusing on Smads and receptors. The first obvious observation is that the TGF-*β *pathway genes do not exist in protozoans but are universally present in metazoans. This leads to the first important conclusion that the TGF-*β *pathway genes evolved rapidly and to a high degree of complexity with the first known animal species. Table 1 provides an overview of the pathway content in high-coverage genomes under study.

**Table 1:** TGF-*β *pathway gene content across the animal taxa.

	Data source	Smad	Receptor	BAMBI (*)	Chordin Family	Gremlin family
*Homo sapiens*	E/TF5	8	13^Ω^	1	7	5

*Pan troglodytes*	E/TF5	8	12	1	7	4

*Macaca mulatta*	E/TF5	8	13^Ω^	1	5	5

*Mus musculus*	E/TF5	8	12	1	7	5

*Rattus norvegicus*	E/TF5	8	13^Ω^	1	6	4

*Canis domesticus*	E/TF5	8	9	1	7	5

*Gallus gallus*	E/TF5	7	11	1	6	3

*Danio rerio*	E/TF5	12^1^	18	1	6	6

*Oryzias latipes*	E/TF5	12^1^	15	1	4	5

*Takifugu rubripes*	E/TF5	13^1^	21	1	7	4

*Tetraodon nigroviridis*	E/TF5	14 ^1^	20	1	9	2

*Ciona savignyi*	E/TF5	5‡	6	--	2	1

*Ciona intestinalis*	E/TF5	5‡‡	7	--	3	1

*Drosophila melanogaster*	FB	4	5	--	1	1

*Apis mellifera*	E/TF5	4	5	--	1	1

*C. elegans, briggsae and remanei*	WB	6	3	--	1	1

*Capitella sp. I*	J	4	5	1	4	1

*Helobdella robusta*	J	4‡‡‡	5	--	--	2

*Lottia gigantea*	J	4	5	1	4	1

*Trichoplax adhaerens*	J	4	4	--	1	1

*Protozoans (Monosiga brevicollis, Volvox carteri, Naegleria gruberi)*	J	--	--	--	--	--

^1 ^For details, see Figure 3

(*) Inhibitory co-receptor BAMBI, although probably present in Urbilateria, appears to be frequently lost, such as in Ecdysozoa, tunicates, and *Helobdella*

‡ Species-specific TGF-*β*-R-Smad duplication (see also Figure S1)

‡‡ Species-specific Co-Smad duplication (see also Figure S1)

‡‡‡ Leech *Helobdella *has a modified pathway: with an additional Co-Smad (Figure S3, [see Additional file 3]) and a distinct set of ligand traps

^Ω ^Including a retrogene of BMPR1A of unverified functionality: ENSG00000185932 (*H. sapiens*, 337 aa, intracellular domains only), ENSMMUG00000031530 (*M. mulatta*, 211 aa, some intracellular domains), ENSRNOG00000011012 (*R. norvegicus*, 529 aa, all domains)

The table indicates the numbers of genes in each family.

Data sources: Ensembl 46 (**E**), TreeFam 5 (**TF5**), JGI (**J**), WormBase (**WB**), FlyBase (**FB**)

### Smads and receptors in Bilateria – point of divergence (POD) analysis

**Figure 1:**
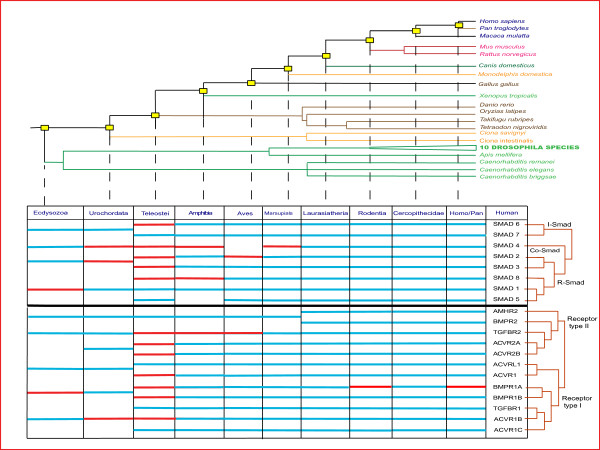
**Evolution of Smads and TGF-*β *receptors in Bilateria**. The species tree, not-to-scale, displays the phylogenetic relationship between humans and the other species, using the monophyletic Ecdysozoa hypothesis. Each point of divergence (POD) group joins together species which share the same ancestor with the evolutionary line leading to humans. POD nodes are marked by yellow boxes. Please, note that PODs differ in strength of available supporting evidence (shown by the species tree). The gene table, at the bottom, describes the relationship between human Smads or receptors (right column of the table, and the adjacent gene trees) and orthologs in POD groups. POD groups are described in the top row of the table, and linked by discontinuous lines to respective POD nodes on the above-mentioned species tree. Inside the cells of the table: blue lines represent one to one orthology (all species of the POD group); red lines represent one (human) to two or more orthology (at least one species of the POD group). Finally, an empty cell signifies a failure to identify an ortholog within a given POD group.

As a general rule, three functional classes of Smads (R-, Co- and I-Smads) are present in all extant species and the reconstructed ancestral genomes. At least one type II receptor and multiple type I receptors can be detected, and the ancestral bilaterian repertoire can be inferred as consisting of two type II receptors and three type I receptors. Detailed observations are provided below, starting with the oldest point of divergence (Figure 1, Table 2, Figure S1 [see Additional file 1], Figure S2 [see Additional file 2]).

**Table 2:** Species codes for Figures S1, S2 (SWcodes).

**Species**	***SWcode***
Apis mellifera	APIME

Danio rerio	BRARE

Caenorhabditis briggsae	CAEBR

Caenorhabditis elegans	CAEEL

Caenorhabditis remanei	CAERE

Canis familiaris	CANFA

Gallus gallus	GALGA

Ciona intestinalis	CIOIN

Ciona savignyi	CIOSA

Drosophila ananassae	DROAN

Drosophila grimshawi	DROGR

Drosophila melanogaster	DROME

Drosophila mojavensis	DROMO

Drosophila persimilis	DROPE

Drosophila sechellia	DROSE

Drosophila simulans	DROSI

Drosophila virilis	DROVI

Drosophila willistoni	DROWI

Drosophila yakuba	DROYA

Fugu rubripes	FUGRU

Homo sapiens	HOMSA

Macaca mulatta	MACMU

Monodelphis domestica	MONDO

Mus musculus	MUSMU

Ornithorhynchus anatinus	ORNAN

Oryzias latipes	ORYLA

Pan troglodytes	PANTR

Rattus norvegicus	RATNO

Tetraodon nigroviridis	TETNG

Xenopus tropicalis	XENTR

#### Ecdysozoan POD

**Figure 2:**
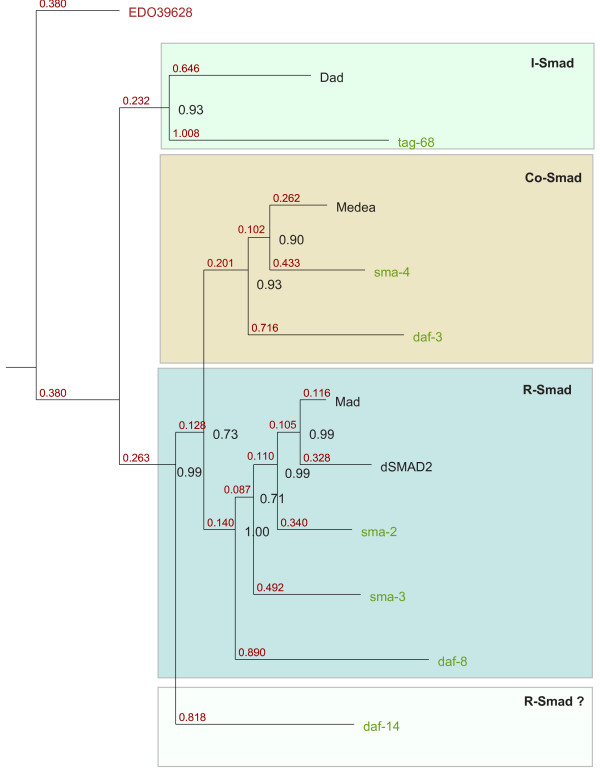
**Amino-acid Bayesian tree of Smads focusing on worm and fly proteins**. The four canonical fly Smads: Mad, dSmad2, dSmad4, and Dad (in black) define the three functional classes of Smads: receptor Smads, Co-Smads and inhibitory Smads. Worm Smads are shown in pale green. Branch lengths are shown in red. Node probabilities are shown in black. The tree is rooted using the *N. vectensis *I-Smad: EDO39628 (in red).

Two R-Smads (one TGF-*β *and one BMP), one Co-Smad and one I-Smad are consistently present in 10 *Drosophila *species, and *Apis mellifera*, and thus can be inferred to have existed in the ancestral genome of the Ecdysozoan POD. *Drosophila *species and *Apis mellifera *also contain two type II receptors and three type I receptors. Nematode genomes contain additional diverged Smads (dauer pathway Smads) but these were excluded from Figure 1 and Figure S1 [see Additional file 1] and analysed separately, because of the special evolutionary status of the dauer pathway (Figure 2).

#### Urochordate PODs

Two sea squirts (*Ciona intestinalis*, *Ciona savignyi*) possess at least two R-Smads (one TGF-*β *and one BMP), one Co-Smad and one I-Smad. Additional Smads can be detected, but these do not cluster with Smads observed in the Vertebrata, and thus represent lineage-specific duplications absent in the genome of the ancestral vertebrate. The ancestral bilaterian TGF-*β *receptor repertoire is expanded to three type II receptors: this is the first example of a bilaterian TGF-*β *receptor duplication, mapping to Chordates in Figure S2 [see Additional file 2], which is propagated through vertebrates.

#### Teleost fish POD

The POD of the teleosts is the first vertebrate POD and also the first POD which can be inferred to possess all eight subtypes of Smads present in extant mammalian genomes (five R-Smads, a Co-Smad and two I-Smads). Additional lineage-specific R-, Co- and I-Smads could also be detected in extant teleost fishes. This stimulated further detailed analysis of teleost fish sequences (see below). All type II and type I receptors have been duplicated, in many cases multiple times (Figure S2 [see Additional file 2]). Some of the progeny genes are common to all vertebrates, several are unique to teleost fishes, and a few are species-specific.

#### Amphibian POD

Amphibians are represented only by one genome – *Xenopus tropicalis*. *Xenopus laevis *was not used, as this species is now widely regarded as tetraploid. *Xenopus tropicalis *possesses a distinct set of nine Smads, with two Co-Smads, two genes for Smad8, and no ortholog of Smad5. The additional Co-Smad does not appear to be a lineage-specific duplication, as it groups with added genes in teleost fishes, suggesting that it may represent a gene deriving directly from the 2R event, lost in other vertebrates.

#### Aves POD

Similar to mammals, the single available avian genome (*Gallus gallus*) contains genes for all five R-Smads, two I-Smads, and five type II and seven type I receptors. Curiously, no Co-Smad was detected in the chicken genome (Figure S1). Manual querying of the ENSEMBL database annotation of the chicken genome (WASHUC2) confirmed that there are no available Co-Smad gene predictions. However, this is a genomic artifact. A representative chicken Co-Smad cluster, Gga.28805 containing 24 EST sequences, was found within the NCBI UniGene collection [43]. Furthermore, examination of synteny with human revealed a large missing sequence region in the chicken genome, which includes orthologs of the extensive gene neighbourhood of the human Co-Smad. This example underlines the need for caution in interpretation of putative losses suggested by genome sequences from individual species.

#### Mammalian PODs (Marsupials, Laurasiatheria, Rodentia, Cercopithecidae, Pan, Homo)

All extant placental mammalian genomes consistently contain a well-characterized set of eight classic mammalian Smads. An additional diverged Co-Smad sequence (ENSMODT00000007722.2) was also detected in the marsupial mammal *Monodelphis domestica*. Interesting observations can be made regarding alternative splicing of the TGF-*β *pathway genes in the mammals. For example, alternative splicing of Smad2 and Smad8, inferred from dbEST, can be traced back to the origin of vertebrates, suggesting a profound functional significance (manual datamining of Ensembl, data not shown). The anti-Mullerian hormone type II receptor (AMHR2) is developed in placental mammals, expanding the receptor repertoire to five type II and seven type I receptors. Retroposed copies of BMPR1A, of unknown functional significance, can also be detected in primates and rodents (Table 1).

### Origin of dauer pathway Smads: duplication, neo-functionalization and accelerated evolution

The phylogenetic relationship between *D. melanogaster*, and *C. elegans *Smads was investigated in further detail (Figure 2). In *C. elegans*, there exist a set of Smads controlling the Sma/Mab pathway (*sma-2*, *sma-3*, *sma-4 *– henceforth collectively termed spSmads), and a set of Smads of the dauer pathway (*daf-3*, *daf-8*, *daf-14 *– henceforth collectively termed dpSmads) [44,45] that were all consistently detected. Functionality of one additional gene *tag-68 *has not been established. Our sequence tree (Figure 2) differs significantly from previously published trees [17,46] in which dpSmads cluster together, not allowing for resolution into proper functional classes or reconstruction of evolutionary origins. Comparison of branch lengths indicates that all dpSmads have been evolving much faster than their counterparts in the Sma/Mab pathway (Figure 2) – a finding suggestive of positive selection acting upon dpSmads. Indeed, accelerated protein sequence change is confirmed by the analysis of Ka/Ks ratios between pairs of orthologs in *C. briggsae *and *C. elegans *(Table 3). Accordingly, all Ka/Ks ratios for known dauer pathway genes in this comparison are higher than ratios for the remaining genes. The average values are 0.72 and 0.16, respectively.

**Table 3:** TGF-*β *pathways of Rhabditoid nematodes.

		***Caenorhabditis elegans***	***Caenorhabditis briggsae***			
						
	**Sub-pathway**	genomic location exon number	Genomic location exon number	Ks*	**Ka**	**Ka/Ks**
**LIGANDS**	**Axon guidance**	*UNC-129* *IV:9005..9003 kbp* *5 exons*	*CBG21741* *IV:4144..4151 kbp* *5 exons*	0.52	0.07	**0.13**
	
		*TIG-2* *V:4729..4726 kbp* *8 exons*	*CBG08804* *V:1854..1850 kbp* *6 exons*	0.56	0.12	**0.21**
	
	***Sma/Mab *pathway**	*DBL-1* *V:6757..6760 kbp* *8 exons*	*CBG19011* *V:9985..9986 kbp* *8 exons*	0.34	0.08	**0.24**
	
	**dauer** **pathway**	*daf-7* *III:811..813 kbp* *5 exons*	*CBG24910* *unassigned* *5 exons*	0.57	0.4	**0.7**

**RECEPTORS**	***Sma/Mab*** **pathway**	*sma-6 *(type I) *II:6324..6327 kbp* 12 exons	*CBG02627* *II:8576..8573 kbp* *11 exons*	0.57	0.15	**0.26**
	
	**dauer** **pathway**	*daf-4 *(type II) *III:5624..5632 kbp* *11 exons*	*CBG08963* *III:3144..3152 kbp* *10 exons*	0.62	0.38	**0.61**
		
		*daf-1 *(type I) *IV:132..138 kbp* *9 exons*	*CBG01651* *IV:10179..10176 kbp* *9 exons*	0.63	0.44	**0.7**

**SMADS**	***Sma/Mab*** **pathway**	*sma-2* *(overlaps with ZK370.8)* *III:8749..8756 kbp* *10 exons*	*CBG06922* *(overlaps with 2 other genes on the opposite strand)* *III:9128..9114 kbp* *7 exons*	0.47	0.02	**0.04**
		
		*sma-3* *III:6863..6860 kbp* *12 exons*	*CBG16541* *III:6052..6058 kbp* *12 exons*	0.51	0.05	**0.1**
		
		*sma-4 *(short form present) *III:5816..5820 kbp* *12 exons*	*CBG09090* *III:2679..2675 kbp* *11 exons*	0.52	0.13	**0.25**
	
	**dauer** **pathway**	*daf-8* *I:8587..8584 kbp* *6 exons*	*CBG12513* *I:7282..7287 kbp* *6 exons*	0.63	0.42	**0.66**
		
		*daf-14* *IV:10253..10255 kbp* *5 exons*	*CBG04415* *IV:11282..11277 kbp* *7 exons*	0.56	0.58	**1.04**
		
		*daf-3* *X:825..817 kbp* *15 exons*	*CBG08108* *X:222..227 kbp* *14 exons*	0.63	0.4	**0.63**
	
		*tag-68* *I:10501..10505 kbp* *9 exons*	*CBG02231* *I:5999..6001 kbp* *9 exons*	0.42	0.01	**0.02**

**SKI**	**dauer** **pathway**	*daf-5* *II:14037..14033 kbp* *5 exons*	*CBG20832* *II:10836..10831 kbp* *6 exons*	0.62	0.44	**0.71**

Comparison of TGF-*β *ligands, receptors and Smads in *C. elegans *and *C. briggsae *reveals conservation of exon number and chromosomal location for both the Sma/Mab and dauer pathways. However, high Ka/Ks ratios for genes of the dauer pathway (underlined) indicate that it evolved faster since the two species diverged. Data retrieved from WormBase (v. WS178).

* modified Nei-Gojobori (p-distance) model with pairwise deletion and assuming transition/transversion ratio of 2.

#### TGF-*β *pathway gene duplication in teleost fishes

**Figure 3:**
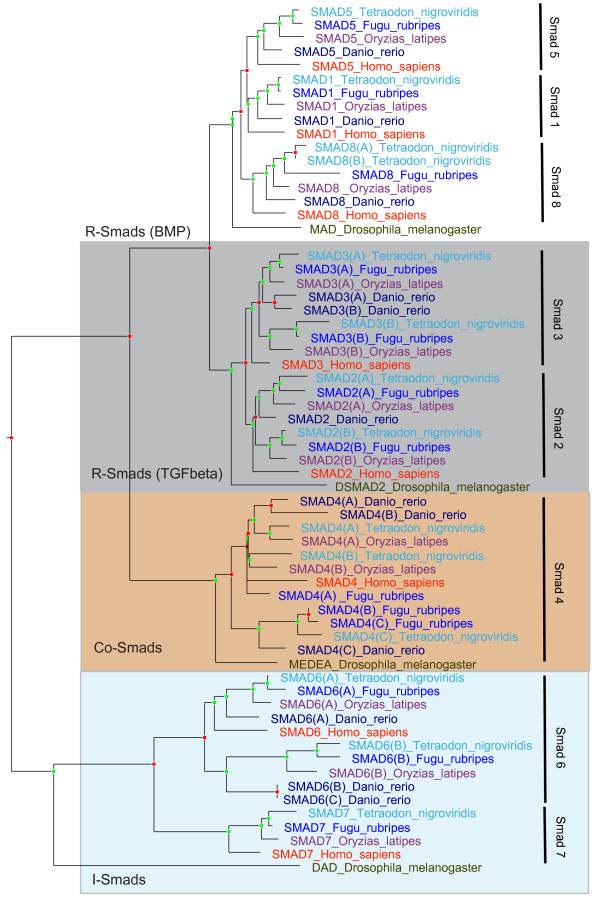
**Multiple additional Smads are present in teleost fishes**. Letters in brackets signify additional teleost fish Smads (co-orthologs in relation to human genes). Fly genes are also shown for comparison. Table 5 lists accession numbers for the relevant genes. The tree is produced using TreeBeST and rooted on time. Red boxes signify duplication nodes, while green boxes signify speciation nodes (inferred using the speciation and duplication inference algorithm).

We have also analyzed the Smads present in zebrafish (*Danio rerio*), medaka (*Oryzias latipes*), fugu (*Takifugu rubripes*) and the green spotted puffer (*Tetraodon nigroviridis*), in comparison with eight human genes representative of vertebrates (Figure 3, Table 5). It is clear that Smads underwent duplications early in teleost fishes, followed by additional lineage-specific duplications. Interestingly, two of the additional Smad2 genes in Tetraodontidae possess a non-classic protein domain: GSTENT00008463001 and SINFRUT00000172868 are predicted to harbour the haem peroxidase domain (IPR002016), which might be utilised in signalling response to oxidative stress. Additional lineage-specific duplications of TGF-*β *receptors can also be detected in these teleost fish species (Figure S2, [see Additional file 2]). What types of novel functions are linked with multiple duplicated Smads and TGF-*β *pathway receptors in teleost fishes remains to be elucidated.

#### Phylogenetic analyses in basal metazoans and Lophotrochozoans

**Figure 4:**
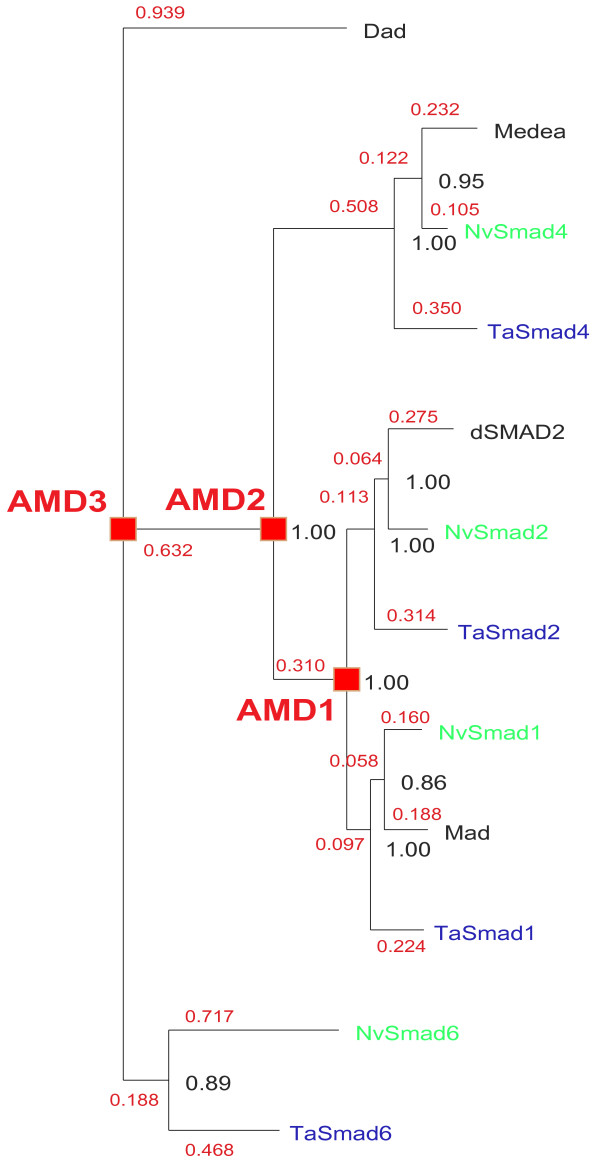
**Basal metazoan repertoire of Smads**. *Trichoplax adhaerens *(prefix Ta – in blue), *Nematostella vectensis *(prefix Nv – in green) and fly proteins (Dad, Medea, dSmad2 and Mad) are shown. The Bayesian tree reveals ancestral metazoan duplications (AMD1, 2 and 3) of the hypothetical single primeval common mediator/receptor activated Smad – note high probability values for all the nodes. *N. vectensis *sequences were retrieved from GenBank: NvSMAD1 (EDO47037), NvSMAD2 (EDO39594), NvSMAD4 (EDO31382), and NvSMAD6 (EDO39628). The tree is rooted using Dad. Branch lengths are shown in red. Node probabilities are shown in black.

**Figure 5:**
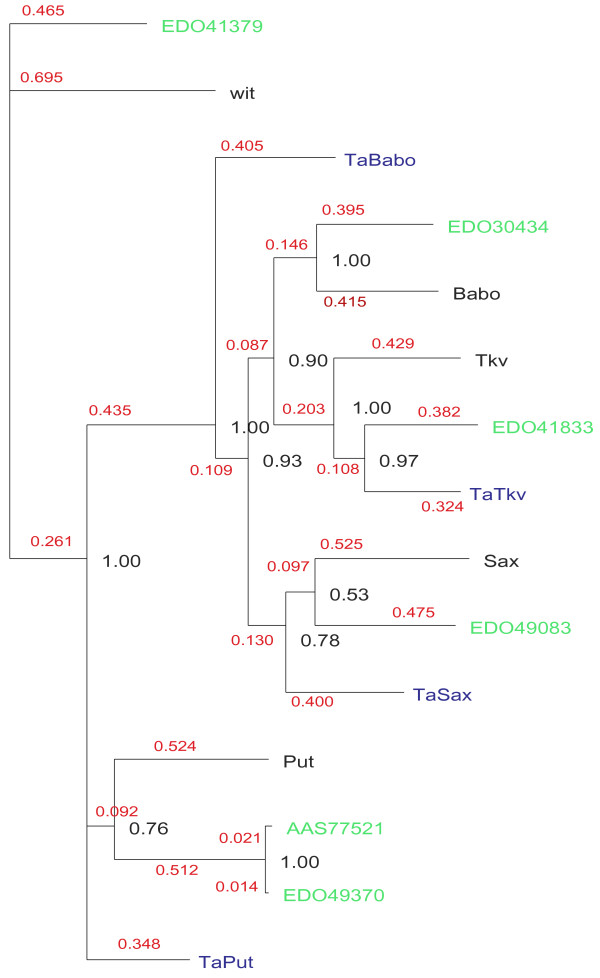
**Amino-acid Bayesian tree showing basal metazoan repertoire of type II/I receptors**. *Trichoplax adhaerens *(prefix Ta – in blue), *Nematostella vectensis *(in green) and fly proteins (Babo, Tkv, Sax, Put, wit) are shown. *N. vectensis *sequences were retrieved from GenBank: type I receptors – EDO30434, EDO41833, EDO49083; type II receptors – EDO41379, and EDO49370 (with splice variant AAS77521). The tree is rooted using EDO41379. Branch lengths are shown in red. Node probabilities are shown in black.

The tree in Figure 4 shows the repertoire of Smads in *Nematostella vectensis *and *Trichoplax adhaerens*, in connection with the reconstruction of ancestral metazoan duplications which resulted in the formation of a complete signalling pathway (including two types of R-Smads, the Co-Smad, and the I-Smad negative feedback loop) in these early diverging animals. It is also worth noting that *Nematostella *and *Trichoplax *contain genes for both receptor classes: type I and type II (Figure 5). However, *Trichoplax*, unlike *Nematostella*, does not appear to harbour an ortholog of wit: TaPut is the only type II receptor found in *Trichoplax *and is likely to correspond to the ancestral type II receptor. Furthermore, while TaSax and TaTkv are clear orthologs of corresponding fly genes, TaBabo branches out deeper in the tree and may correspond to the ancestral type I receptor.

The Bayesian tree in Figure S3 [see Additional file 3] (Dad displayed as outgroup) demonstrates that the familiar pattern of four Smads grouped into three functional classes can be also observed in comparatively poorly investigated Lophotrochozoans (*Capitella sp. I, Helobdella robusta*, and *Lottia gigantea*). The Bayesian tree in Figure S4 [see Additional file 4] (Dad displayed as outgroup) shows two *Amphimedon *R-Smads (AqSmad1 and AqSmad2) which are the only Smads we have detected in genomic traces available for this demosponge. Species codes can be accessed in Table 2.

## Discussion

The growing number of sequenced genomes provides a relatively wide coverage of the animal genome space. This makes it possible to reconstruct ancestral developmental signalling pathways, and to retrace the ancient evolutionary events which led to their emergence and modulation, in particular gene duplications, instances of sub- and neo-functionalization, and gene losses. Herein, we focus on the gene set constituting the fundamental building blocks of a major component of the animal developmental toolkit – the TGF-*β *pathway.

We have examined in detail the gene content of the TGF-*β *pathway in extant genomes of different metazoan phyla, where high-coverage genomic data are available (Table 1). Smads are of particular interest, as they constitute the core engine of the TGF-*β *signal transduction machinery. We have estimated the origin of different types of Smads by examining extant genomes and inferring ancestral genes (Point of Divergence analysis – Figure 1 summarises Figure S1 [see Additional file 1]). We justify somewhat anthropocentric approach of the POD analysis by the high significance of the TGF-*β *pathway in human health and disease, which drives substantial proportion of research in the field. On the lineage of PODs leading to human, the Smads clearly appear to have gone through a major wave of duplications, fitting well with the 2R hypothesis of two-fold genome duplication at the base of vertebrates [47-50]. Additional duplications occurred along the teleost fish lineage, in congruence with the hypothesis of a teleost fish-specific genome duplication – FSGD [51,52]. Diversification of type I and type II receptors has also followed the course agreeable with the 2R hypothesis, with multiple additional duplications in teleost fishes (Figure S2, [see Additional file 2]).

POD analysis (Figure 1) shows that the core pathway (both receptors and Smads) expanded dramatically and permanently at the base of vertebrates. Table 1 demonstrates that this expansion correlates well with the increase of complexity of regulatory networks associated with the extended pathway, such as ligand traps of the chordin and gremlin family. The same is true of many transcriptional co-activators, and target genes – particularly those in the concurrently developed active immune system, as well as the endocytic regulators that control Smad signalling, SARA and endofin, which emerged through the duplication of a single ancestral gene (data not shown).

Analysis of the *C. elegans *genome revealed atypical Smads belonging to Sma/Mab and dauer pathways. Our phylogenetic tree indicates that *daf-8 *is an R-Smad, *daf-3 *a Co-Smad and *Tag-68 *an I-Smad (Figure 2). *Sma-2 *and *sma-3 *are likely duplicates of the ancestral BMP R-Smad, as they both contain the characteristic RQDVTS motif of the L3 loop. Conversely, *daf-8 *and *daf-14 *might be duplicates of the ancestral TGF-*β *R-Smad, although *daf-14 *is too divergent to allow firm conclusions. *Sma-4 *and *daf-3 *share a similar pattern of multiple splice variants, which together with the tree topology suggests that they derive from the ancestral Co-Smad via a gene duplication event. Comparative analysis revealed that Sma/Mab and dauer pathway content is identical between *C. elegans *and *C. briggsae*, with strong conservation of the overall gene structure and synteny (Table 3). This proves that all the relevant genes already existed in the last common ancestor of the two Rhabditoid species. Although similar in morphology, *C. briggsae *and *C. elegans *are rather distant relatives in evolutionary time: the two species split roughly 100 million years ago [53]. Analysis of lengths of protein branches (Figure 2) is indicative of accelerated evolution of *daf-3*, *8 *and *14*. Additionally, analysis of Ka/Ks ratios between pairs of orthologs in *C. briggsae *and *C. elegans *suggests that the dauer pathway evolved faster since the two species diverged (Table 3). The average Ka/Ks ratio for dauer pathway orthologs is 0.72 versus 0.16 for non-dauer TGF-*β *pathway genes. Thus, the initial duplications and neo-functionalization occurred early in nematode evolution, but have been followed by further change in separate Rhabditoid lineages, as different species experienced slightly different selection pressures for entry and persistence in their dauer forms. For example, *C. elegans*, unlike *C. briggsae*, is strongly induced to form dauers at temperatures higher than 26°C [54]. Overall, the dauer pathway represents an interesting example of rapidly evolving pathway neo-functionalization, developed as a lineage-specific adaptation towards the colonization of the environmental niche of the soil.

The crucial question about the taxonomic origin of the TGF-*β *pathway has not been categorically answered yet. Herein, we have identified TGF-*β *pathway components in *T. adhaerens*, the representative of the early diverging phylum Placozoa, and the demosponge *Amphimedon queenslandica *[55]. Choanoflagellata are the closest unicellular relatives of animals [32] and possess some genes linked to metazoan development, for example a receptor tyrosine kinase – MBRTK1 [56]. However, we have not been able to detect Smads, TGF-*β *receptors, ligands, SARA, chordin or gremlin in the genome of the unicellular choanoflagellate *M. brevicollis *[33], or more distantly related protozoans *Volvox carteri *and *Naegleria gruberi*. This indicates that the appearance of the TGF-*β *pathway was intrinsically linked to the emergence of earliest animals, and the pathway may thus be regarded as a key feature of the metazoan life forms. It is also rather striking that such an early diverging animal as *Trichoplax *already possesses the complete functional pathway, including multiple Smads, receptors, and ligands, as well as orthologs of chordin, gremlin and SARA.

We hypothesize that the single primeval common mediator/receptor activated Smad functioned as a homo-dimer (or homo-trimer), and possessed the universal functionality of R-Smads and the Co-Smad; i.e. it could be phosphorylated by the receptor/ligand complex, shuttle to the nucleus, interact with transcriptional co-activators via the MH2 domain and bind DNA via the MH1 domain. As the number of ligands and receptors grew, the primeval Smad duplicated and, through sub-functionalization, gave rise to two separate R-Smads which from then on interact with non-overlapping sets of receptors (Figure 4: ancestral metazoan duplication – AMD 1; Table 4). One of the R-Smads duplicated again (Figure 4: AMD 2) giving rise to a Co-Smad which enhanced the set of regulatory protein interactions, and possibly provided a way of integrating the signals from the two R-Smad channels through competition for the available pool of Co-Smad molecules. The critical role of Co-Smad bioavailability is also suggested by its low duplicability; in the great majority of species there is only one Co-Smad (Figure 1). *Xenopus laevis *is the notable exception having two genes XSMad4a and XSmad4b [57,58], but they are differentially expressed both in embryos and adult tissues. The fast diverging I-Smad was the last addition to the pathway (Figure 4: AMD 3). It neofunctionalized to create a controlling negative feedback loop; I-Smad transcription is induced by the pathway, the protein can bind the activated receptor complex, but lacking a terminal phosphorylation motif it does not propagate the signal. Since it is not being used, over time the MH1 of the I-Smad converted to a vestigial domain. It will be interesting to see if future genome projects of basal animals and closest extant unicellular relatives of animals [59] will provide a proof of our single Smad hypothesis.

**Table 4:** Receptor-Smad specificity.

**Receptor II**	**Receptor I**	**R-Smad**	**Ligand family**
AMHR2	ACVR1	Smad1,5,8	AMH
	BMPR1A	Smad1,5,8	
	BMPR1B	Smad1,5,8	

BMPR2	BMPR1A	Smad1,5,8	BMP2/4/6/7/9/10/13/15
	BMPR1B	Smad1,5,8	
	ACVR1	Smad1,5,8	
	ACVRL1	Smad1,5,8	
			
	TGFBR1	Smad2,3	GDF9

TGFBR2	TGFBR1	Smad2,3	TGFB1/2/3

ACVR2A	ACVR1	Smad1,5,8	BMP4/5/7/9/10/14/15
	BMPR1A	Smad1,5,8	
	BMPR1B	Smad1,5,8	
	ACVRL1	Smad1,5,8	
			
	ACVR1B	Smad2,3	Nodal, GDF1/11, ActA/B/AB, InhA/B/C
	ACVR1C	Smad2,3	

ACVR2B	ACVR1	Smad1,5,8	BMP4/7/14
	BMPR1A	Smad1,5,8	
	BMPR1B	Smad1,5,8	
			
	ACVR1B	Smad2,3	Nodal, GDF1/3/8/11, ActA/B/AB, InhA/B/C
	ACVR1C	Smad2,3	
	TGFBR1	Smad2,3	

Mammalian TGF-*β *type II receptors are listed in the first row. Type I receptors which make functional complexes with each type II receptor are then listed, followed by the R-Smads that the type I receptor activates. The ligands activating each receptor-Smad group are then listed in a cumulative fashion.

**Table 5:** Accession numbers for Figure 3.

**Smad**	**Accession number**
**Smad7**	ENST00000262158, ENSORLT00000007300, SINFRUT00000168711, GSTENT00034726001

**Smad6**	ENSDART00000075213, ENSDART00000014508, ENSORLT00000002768, SINFRUT00000132241, GSTENT00018098001, ENST00000288840, ENSDART00000049006, ENSORLT00000008573, SINFRUT00000171835, GSTENT00016030001

**Smad4**	ENSDART00000047862, GSTENT00008799001, SINFRUT00000174976, SINFRUT00000173081, SINFRUT00000170229, ENST00000342988, ENSORLT00000009648, GSTENT00017220001, ENSORLT00000006329, GSTENT00004746001, ENSDART00000048201, ENSDART00000035478

**Smad2**	ENST00000356825, ENSORLT00000019644, SINFRUT00000167872, GSTENT00021584001, ENSDART00000003587, ENSORLT00000014832, SINFRUT00000172868, GSTENT00008463001

**Smad3**	ENST00000327367, ENSORLT00000008549, SINFRUT00000175526, GSTENT00016035001, ENSDART00000045374, ENSDART00000043455, ENSORLT00000002749, SINFRUT00000167684, GSTENT00018100001

**Smad8**	ENST00000379826, ENSDART00000028618, ENSORLT00000004945, SINFRUT00000183098, GSTENT00018869001, GSTENT00002259001

**Smad1**	ENST00000302085, ENSDART00000033566, ENSORLT00000009248, SINFRUT00000133755, GSTENT00032865001

**Smad5**	ENST00000231589, ENSDART00000054175, ENSORLT00000011780, SINFRUT00000175729, GSTENT00005726001

## Conclusion

The emergence of the TGF-*β *pathway coincided with appearance of the first animal species, and was most likely linked with duplications of the single primeval common mediator/receptor activated Smad. This resulted in the creation of the ancestral eumetazoan repertoire of four Smads, forming the basis of the pathway in the Placozoa, the Cnidaria, the Arthropoda, and in the Lophotrochozoa. After application of a formal speciation and duplication inference algorithm, we conclude that the diversification of Smads and receptors in chordates is parsimoniously explained according to the 2R model, with additional rounds of duplications in teleost fishes. The Nematoda posses a heavily modified pathway which evolution has been marked by accelerated sequence change.

Our multi-genome comparison and ancestral inference approach has implications extending beyond the TGF-*β *pathway. Origins of other developmental signalling pathways, for example Hox and hedgehog, are also being investigated using phylogenomic approaches [60,61]. Results obtained for all developmental signalling pathways should be integrated and compared with paleontological records and molecular clock data, to identify the molecular nature and timing of all major changes in the shared animal developmental toolkit [62], including those which gave rise to vertebrate innovations [63].

## Methods

### TGF-*β *pathway gene content across the animal taxa

Table 1 presents the number of paralogous genes in metazoan genomes, where high-coverage sequence data and reliable gene predictions are available.

### Reconstructing Smad content in ancestral species

Known human Smads and TGF-*β *receptor proteins were used for a BLASTP search against a collection of proteomes predicted for high-coverage sequenced genomes, providing as wide as possible coverage of the animal kingdom. BLASTP parameters were first calibrated to yield searches of optimal sensitivity and specificity using human and mouse genomes (where the identity of relevant genes is well known) and verified using more distantly related animal genomes, through manual inspection of hits and alignments (to avoid, for example, non-specific hits to the kinase domain of the receptors). The following E-value cut-offs were used: 10e-30 for receptors and 10e-20 for Smads.

It is important to notice that searches against the collection of proteomes were unbiased by the identity of species used as the starting point. No additional genes can be identified when searching with *D. melanogaster, Nematostella*, *Trichoplax *or *Lottia gigantea *Smads and receptors. In fact, these proteins are so well conserved in sequence that searches starting with genes originating from different phyla are essentially equivalent. For example, when Smads and receptors from human, *D. melanogaster, Nematostella*, *Trichoplax *or *Lottia gigantea *were used as queries against their proteomes as well as those of *Xenopus tropicalis*, *Monodelphis domestica*, *Danio rerio*, *Ciona savignyi*, and *Caenorhabditis elegans*, identical lists of hits were obtained (except that query using *Trichoplax *receptors did not detect one gene in human, *M. domestica *and *X. tropicalis*).

The lists of homologs were further filtered, in order to include only those proteins which contained an exemplary Pfam domain [64]: MH2 for Smads; and any of the following for TGF-*β *receptors: an activin-type I/II receptor domain, a TGF-*β *receptor domain, or a TGF-*β*-GS motif for type I receptors [see Additional file 5]. Presence of the terminal phosphorylation motif (SSxS) was also verified in case of R-Smads. Multiple sequence alignments were performed using Muscle [65].

### Smads and receptors in Bilateria – point of divergence (POD) analysis

The ancestral state of the pathway was estimated by analyzing the orthology relationship between the human proteins and the proteins in the genomes of extant species within collective POD groups (Figure 1 summarises Figures S1 and S2). Orthology was deduced from phylogenetic trees (through gene/species tree reconciliation). Table 2 lists species codes used in Figures S1 and S2. POD analysis is a graphical shortcut equivalent to manually traversing a gene tree according to a species tree, which facilitates ancestral gene content reconstruction. Additionally, gene duplications and losses were inferred using the speciation and duplication inference algorithm (SDI) [66], modified to work with non-binary species tree.

### Identification of Smads in the genome of the demosponge Amphimedon queenslandica (formerly Reniera sp.)

*Amphimedon *traces were fetched from the NCBI trace archive in May 2008. Low stringency Tblastn query (-E 0.01) with a human R-Smad sequence (Smad2) was used to identify traces with a minimal Smad coding potential. Resulting 383 traces were clipped to avoid low quality 5'- and 3'-termini and assembled into 30 contigs using Cap3 with default parameters [67]. Genewisedb [68] (-splice flat -intron tied -trans -hmmer) invoked with a custom hmm profile compiled from all bilaterian Smad sequences was used to predict putative Smad genes on the 30 contigs. Resulting proteins were checked against the base quality and trace coverage of the underlying contig sequence and validated against Pfam MH1 and MH2 domain models. Based on tree topology, two putative R-Smads were identified (Figure S4, [see Additional file 4], [see Additional file 5]).

### Analysis of the evolutionary rates

Ka and Ks calculations were performed using the modified Nei-Gojobori (p-distance) model [69] with pairwise deletion and assuming transition/transversion ratio of 2 – as implemented in the phylogenetic analysis package Mega 3.1 [70].

### Phylogenetic analyses

We have utilized two approaches to phylogenetic inference to capitalize on advantages offered by different methods. Large-scale trees with sequences from many genomes (termed phylogenomic trees) were produced using particularly suited TreeBeST. Computationally intensive Bayesian method was applied to small-scale trees, including a difficult phylogenetic problem involving worm Smads.

### Phylogenomic trees

Maximum likelihood trees were produced using a fast hill-climbing algorithm which adjusts tree topology and branch lengths simultaneously [71]. Smad and receptor nucleotide sequences were aligned with protein alignment as guide using RevTrans-1.4. The maximum likelihood tree was then merged with a Ks neighbor-joining tree using the TreeBeST [72] phylogenetic engine (to produce Figure S1 [see Additional file 1] and S2 [see Additional file 2]). TreeBeST is part of the TreeSoft project [73], and has been tested extensively against knowledge of biologists, including manual curation, within the TreeFam and Ensembl databases. Trees were rooted on time, and speciation and duplication inference algorithm (SDI), based on the reconciliation of the gene tree with a trusted species tree [66], was used to infer orthology, paralogy, speciation nodes and gene duplication events. However, inferred duplication events with no species intersection support (SIS = 0) were attributed to locally incorrect gene tree topology. ATV was used as a tree viewer [74].

### Bayesian phylogenetic inference

MrBayes3 [75] was used to generate trees with node probabilities in Figures 2, 4, 5, S3 and S4. For these analyses, Metropolis coupling variant of Markov chain Monte Carlo algorithm [76] was run with a mixture of protein evolution models with fixed rate matrices [75], and assuming equal rates, for 100,000 generations, sampling every 100^th ^generation and discarding initial 25% trees (see manual [77]).

## Authors' contributions

LH gathered the data, designed and performed all the analyses, and wrote the manuscript. AM and CHH participated in the study design, provided feedback on results, and contributed to writing the manuscript. LG, SF and CO prepared a pilot version of Figure 1 and were involved in drafting the manuscript. All authors read and approved the final manuscript.

## Supplementary Material

Additional file 1 **Figure S1.** Smad phylogenomic tree (rooted on time). View in magnification (at least 200%), in a pdf viewer such as Acrobat Reader, Adobe Acrobat or kpdf. Click here for file

Additional file 2 **Figure S2.** Receptor phylogenomic tree (rooted on time). View in magnification (at least 200%), in a pdf viewer such as Acrobat Reader, Adobe Acrobat or kpdf. Click here for file

Additional file 3 **Figure S3.** Bayesian phylogenetic tree demonstrates that the familiar pattern of four Smads grouped into three functional classes can be also observed in Lophotrochozoans. The tree is rooted using Dad. Accessions given are JGI gene model numbers. Click here for file

Additional file 4 **Figure S4. **Two R-Smads (AqSmad1 and AqSmad2) have been detected in Amphimedon genomic traces. The tree is rooted using Dad. Click here for file

Additional file 5 **Sequences.** All sequences and alignments are provided in the supplementary archive. Click here for file

## References

[B1] Ohno S (1970). Evolution by Gene and Genome Duplication.

[B2] Lynch M, Conery JS (2000). The evolutionary fate and consequences of duplicate genes. Science.

[B3] Lynch M, O'Hely M, Walsh B, Force A (2001). The probability of preservation of a newly arisen gene duplicate. Genetics.

[B4] Hughes AL (1994). The evolution of functionally novel proteins after gene duplication. Proc R Soc Lond B Biol Sci.

[B5] Force A, Lynch M, Pickett FB, Amores A, Yan YL, Postlethwait J (1999). Preservation of duplicate genes by complementary, degenerative mutations. Genetics.

[B6] Huminiecki L, Wolfe KH (2004). Divergence of spatial gene expression profiles following species-specific gene duplications in human and mouse. Genome Res.

[B7] Khaitovich P, Weiss G, Lachmann M, Hellmann I, Enard W, Muetzel B, Wirkner U, Ansorge W, Paabo S (2004). A neutral model of transcriptome evolution. PLoS Biol.

[B8] Kondrashov FA, Rogozin IB, Wolf YI, Koonin EV (2002). Selection in the evolution of gene duplications. Genome Biol.

[B9] Massague J, Gomis RR (2006). The logic of TGFbeta signaling. FEBS Letters.

[B10] Ten Dijke P, Heldin CH (2006). Smad Signal Transduction: Smads in Proliferation, Differentiation and Disease.

[B11] Heldin CH, Miyazono K, ten Dijke P (1997). TGF-beta signalling from cell membrane to nucleus through SMAD proteins. Nature.

[B12] Vastrik I, D'Eustachio P, Schmidt E, Joshi-Tope G, Gopinath G, Croft D, de Bono B, Gillespie M, Jassal B, Lewis S (2007). Reactome: a knowledge base of biologic pathways and processes. Genome Biology.

[B13] Reactome (REACT_12034 REACT_6844). http://www.reactome.org/.

[B14] Derynck R, (Ed) (2007). The TGF-beta Family.

[B15] Massague J, Seoane J, Wotton D (2005). Smad transcription factors. Genes & Development.

[B16] Wrana JL, Ozdamar B, Le Roy C, Benchabane H, Derynck R, Miyazono K (2007). Signaling Receptors of the TGF-beta Family. The TGF-beta Family.

[B17] Newfeld SJ, Wisotzkey RG, Kumar S (1999). Molecular evolution of a developmental pathway: phylogenetic analyses of transforming growth factor-beta family ligands, receptors and Smad signal transducers. Genetics.

[B18] Derynck R, Miyazono K, Derynck R, Miyazono K (2008). TGF-beta and the TGF-beta Family. The TGF-beta Family.

[B19] Herpin A, Lelong C, Favrel P (2004). Transforming growth factor-beta-related proteins: an ancestral and widespread superfamily of cytokines in metazoans. Developmental and Comparative Immunology.

[B20] Chang C, Derynck R, Miyazono K (2007). Agonists and Antagonists of the TGF-beta Family Ligands. The TGF-beta Family.

[B21] Onichtchouk D, Chen YG, Dosch R, Gawantka V, Delius H, Massague J, Niehrs C (1999). Silencing of TGF-beta signalling by the pseudoreceptor BAMBI. Nature.

[B22] Garcia-Fernandez J, D'Aniello S, Escriva H (2007). Organizing chordates with an organizer. Bioessays.

[B23] Samuel G, Miller D, Saint R (2001). Conservation of a DPP/BMP signaling pathway in the nonbilateral cnidarian Acropora millepora. Evolution & Development.

[B24] Hobmayer B, Rentzsch F, Holstein TW (2001). Identification and expression of HySmad1, a member of the R-Smad family of TGFbeta signal transducers, in the diploblastic metazoan Hydra. Development Genes & Evolution.

[B25] Matus DQ, Thomsen GH, Martindale MQ (2006). Dorso/ventral genes are asymmetrically expressed and involved in germ-layer demarcation during cnidarian gastrulation. Current Biology.

[B26] Matus DQ, Pang K, Marlow H, Dunn CW, Thomsen GH, Martindale MQ (2006). Molecular evidence for deep evolutionary roots of bilaterality in animal development. Proceedings of the National Academy of Sciences of the United States of America.

[B27] Reber-Muller S, Streitwolf-Engel R, Yanze N, Schmid V, Stierwald M, Erb M, Seipel K (2006). BMP2/4 and BMP5-8 in jellyfish development and transdifferentiation. International Journal of Developmental Biology.

[B28] Hayward DC, Samuel G, Pontynen PC, Catmull J, Saint R, Miller DJ, Ball EE (2002). Localized expression of a dpp/BMP2/4 ortholog in a coral embryo. Proceedings of the National Academy of Sciences of the United States of America.

[B29] Rentzsch F, Guder C, Vocke D, Hobmayer B, Holstein TW (2007). An ancient chordin-like gene in organizer formation of Hydra. Proceedings of the National Academy of Sciences of the United States of America.

[B30] Brooke NM, Holland PW (2003). The evolution of multicellularity and early animal genomes. Current Opinion in Genetics & Development.

[B31] Ruiz-Trillo I, Burger G, Holland PW, King N, Lang BF, Roger AJ, Gray MW (2007). The origins of multicellularity: a multi-taxon genome initiative. Trends in Genetics.

[B32] Lang BF, O'Kelly C, Nerad T, Gray MW, Burger G (2002). The closest unicellular relatives of animals. Current Biology.

[B33] King N, Westbrook MJ, Young SL, Kuo A, Abedin M, Chapman J, Fairclough S, Hellsten U, Isogai Y, Letunic I (2008). The genome of the choanoflagellate Monosiga brevicollis and the origin of metazoans. Nature.

[B34] Schierwater B (2005). My favorite animal, Trichoplax adhaerens. Bioessays.

[B35] Voigt O, Collins AG, Pearse VB, Pearse JS, Ender A, Hadrys H, Schierwater B (2004). Placozoa – no longer a phylum of one. Current Biology.

[B36] Dellaporta SL, Xu A, Sagasser S, Jakob W, Moreno MA, Buss LW, Schierwater B (2006). Mitochondrial genome of Trichoplax adhaerens supports Placozoa as the basal lower metazoan phylum. PNAS.

[B37] Leys SP, Rohksar DS, Degnan BM (2005). Sponges. Current Biology.

[B38] Nielsen C (2008). Six major steps in animal evolution: are we derived sponge larvae?. Evolution & Development.

[B39] Patterson GI, Padgett RW (2000). TGF beta-related pathways. Roles in Caenorhabditis elegans development. Trends in Genetics.

[B40] Wood WB, Johnson TE (1994). Aging. Stopping the clock. Current Biology.

[B41] Fitch DH (2005). Evolution: an ecological context for C. elegans. Current Biology.

[B42] Viney ME, Thompson FJ, Crook M (2005). TGF-beta and the evolution of nematode parasitism. International Journal for Parasitology.

[B43] Wheeler DL, Barrett T, Benson DA, Bryant SH, Canese K, Chetvernin V, Church DM, DiCuccio M, Edgar R, Federhen S (2007). Database resources of the National Center for Biotechnology Information. Nucleic Acids Research.

[B44] Savage-Dunn C, Maduzia LL, Zimmerman CM, Roberts AF, Cohen S, Tokarz R, Padgett RW (2003). Genetic screen for small body size mutants in C. elegans reveals many TGFbeta pathway components. Genesis.

[B45] Savage-Dunn C, Tokarz R, Wang H, Cohen S, Giannikas C, Padgett RW (2000). SMA-3 smad has specific and critical functions in DBL-1/SMA-6 TGFbeta-related signaling. Developmental Biology.

[B46] Kloos DU, Choi C, Wingender E (2002). The TGF-beta – Smad network: introducing bioinformatic tools. Trends in Genetics.

[B47] Furlong RF, Holland PW (2002). Were vertebrates octoploid?. Philosophical Transactions of the Royal Society of London – Series B: Biological Sciences.

[B48] Holland PW, Garcia-Fernandez J, Williams NA, Sidow A (1994). Gene duplications and the origins of vertebrate development. Development Supplement.

[B49] Sidow A (1996). Gen(om)e duplications in the evolution of early vertebrates. Current Opinion in Genetics & Development.

[B50] Putnam NH, Butts T, Ferrier DE, Furlong RF, Hellsten U, Kawashima T, Robinson-Rechavi M, Shoguchi E, Terry A, Yu JK (2008). The amphioxus genome and the evolution of the chordate karyotype. Nature.

[B51] Taylor JS, Braasch I, Frickey T, Meyer A, Peer Y Van de (2003). Genome duplication, a trait shared by 22000 species of ray-finned fish. Genome Research.

[B52] Meyer A, Schartl M (1999). Gene and genome duplications in vertebrates: the one-to-four (-to-eight in fish) rule and the evolution of novel gene functions. Current Opinion in Cell Biology.

[B53] Stein LD, Bao Z, Blasiar D, Blumenthal T, Brent MR, Chen N, Chinwalla A, Clarke L, Clee C, Coghlan A (2003). The genome sequence of Caenorhabditis briggsae: a platform for comparative genomics. Plos Biology.

[B54] Inoue TAM, Poon S, Kim HK, Thomas JH, Sternberg PW (2007). Genetic analysis of dauer formation in Caenorhabditis briggsae. Genetics.

[B55] Adamska M, Degnan SM, Green KM, Adamski M, Craigie A, Larroux C, Degnan BM (2007). Wnt and TGF-beta expression in the sponge Amphimedon queenslandica and the origin of metazoan embryonic patterning. PLoS ONE.

[B56] King N, Carroll SB (2001). A receptor tyrosine kinase from choanoflagellates: molecular insights into early animal evolution. Proceedings of the National Academy of Sciences of the United States of America.

[B57] Howell M, Itoh F, Pierreux CE, Valgeirsdottir S, Itoh S, ten Dijke P, Hill CS (1999). Xenopus Smad4beta is the co-Smad component of developmentally regulated transcription factor complexes responsible for induction of early mesodermal genes. Developmental Biology.

[B58] Masuyama N, Hanafusa H, Kusakabe M, Shibuya H, Nishida E (1999). Identification of two Smad4 proteins in Xenopus. Their common and distinct properties. J Biol Chem.

[B59] Ruiz-Trillo I, Burger G, Holland PW, King N, Lang BF, Roger AJ, Gray MW (2007). The origins of multicellularity: a multi-taxon genome initiative. Trends Genet.

[B60] Larroux C, Fahey B, Degnan SM, Adamski M, Rokhsar DS, Degnan BM (2007). The NK homeobox gene cluster predates the origin of Hox genes. Current Biology.

[B61] Adamska M, Matus DQ, Adamski M, Green K, Rokhsar DS, Martindale MQ, Degnan BM (2007). The evolutionary origin of hedgehog proteins. Current Biology.

[B62] Carroll SB, Grenier JK, Weatherbee SD (2005). From DNA to Diversity.

[B63] Shimeld SM, Holland PW (2000). Vertebrate innovations. Proceedings of the National Academy of Sciences of the United States of America.

[B64] Bateman A, Coin L, Durbin R, Finn RD, Hollich V, Griffiths-Jones S, Khanna A, Marshall M, Moxon S, Sonnhammer EL (2004). The Pfam protein families database. Nucleic Acids Research.

[B65] Edgar RC (2004). MUSCLE: a multiple sequence alignment method with reduced time and space complexity. BMC Bioinformatics.

[B66] Zmasek CM, Eddy SR (2001). A simple algorithm to infer gene duplication and speciation events on a gene tree. Bioinformatics.

[B67] Huang X, Madan A (1999). CAP3: A DNA sequence assembly program. Genome Research.

[B68] Birney E, Clamp M, Durbin R (2004). GeneWise and Genomewise. Genome Research.

[B69] Nei M, Gojobori T (1986). Simple methods for estimating the numbers of synonymous and nonsynonymous nucleotide substitutions. Mol Biol Evol.

[B70] Kumar S, Tamura K, Nei M (2004). MEGA3: Integrated software for Molecular Evolutionary Genetics Analysis and sequence alignment. Briefings in Bioinformatics.

[B71] Guindon S, Gascuel O (2003). A simple, fast, and accurate algorithm to estimate large phylogenies by maximum likelihood. Systematic Biology.

[B72] Heng L (2006). Constructing the Treefam Database.

[B73] TreeSoft project. http://sourceforge.net/projects/treesoft/.

[B74] Zmasek CM, Eddy SR (2001). ATV: display and manipulation of annotated phylogenetic trees. Bioinformatics.

[B75] Ronquist F, Huelsenbeck JP (2003). MrBayes 3: Bayesian phylogenetic inference under mixed models. Bioinformatics.

[B76] Huelsenbeck JP, Ronquist F (2001). MRBAYES: Bayesian inference of phylogenetic trees. Bioinformatics.

[B77] MrBayes Manual. http://mrbayes.csit.fsu.edu/manual.php.

[B78] US Department of Energy Joint Genome Institute. http://www.jgi.doe.gov/.

