# Supplementary figures and images for: Emergence, development and diversification of the TGF-β signalling pathway within the animal kingdom
Source: BMC Evol Biol. 2009 Feb 3;9:28. doi: 10.1186/1471-2148-9-28 (PMC2657120; doi:10.1186/1471-2148-9-28)

## R-Smads (TGFbeta)

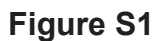

Supplement: Additional file 1 — Figure S1. Smad phylogenomic tree (rooted on time). View in magnification (at least 200%), in a pdf viewer such as Acrobat Reader, Adobe Acrobat or kpdf. [file 1471-2148-9-28-S1.pdf]

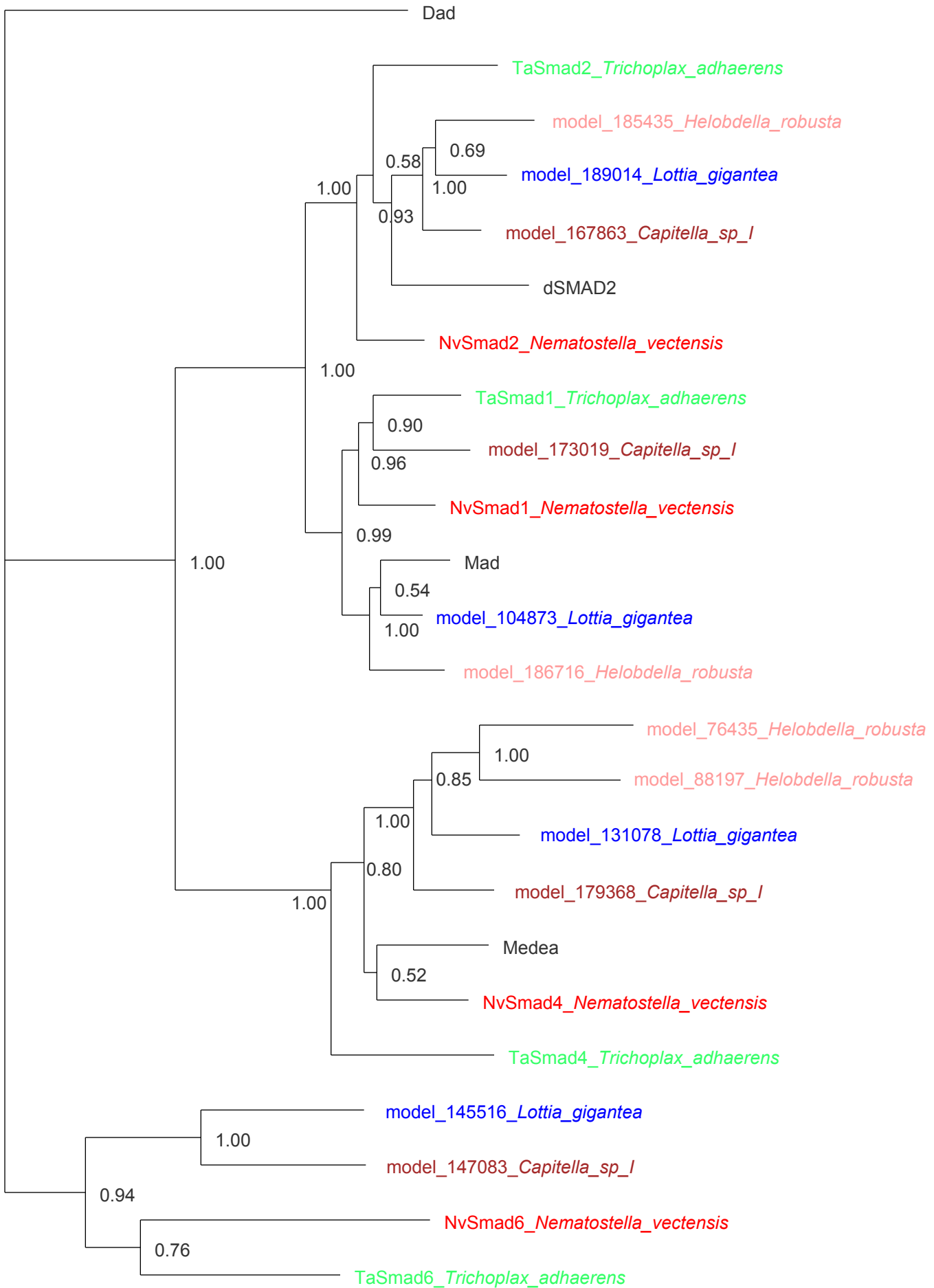

**Figure S3**

Supplement: Additional file 3 — Figure S3. Bayesian phylogenetic tree demonstrates that the familiar pattern of four Smads grouped into three functional classes can be also observed in Lophotrochozoans. The tree is rooted using Dad. Accessions given are JGI gene model numbers. [file 1471-2148-9-28-S3.pdf]

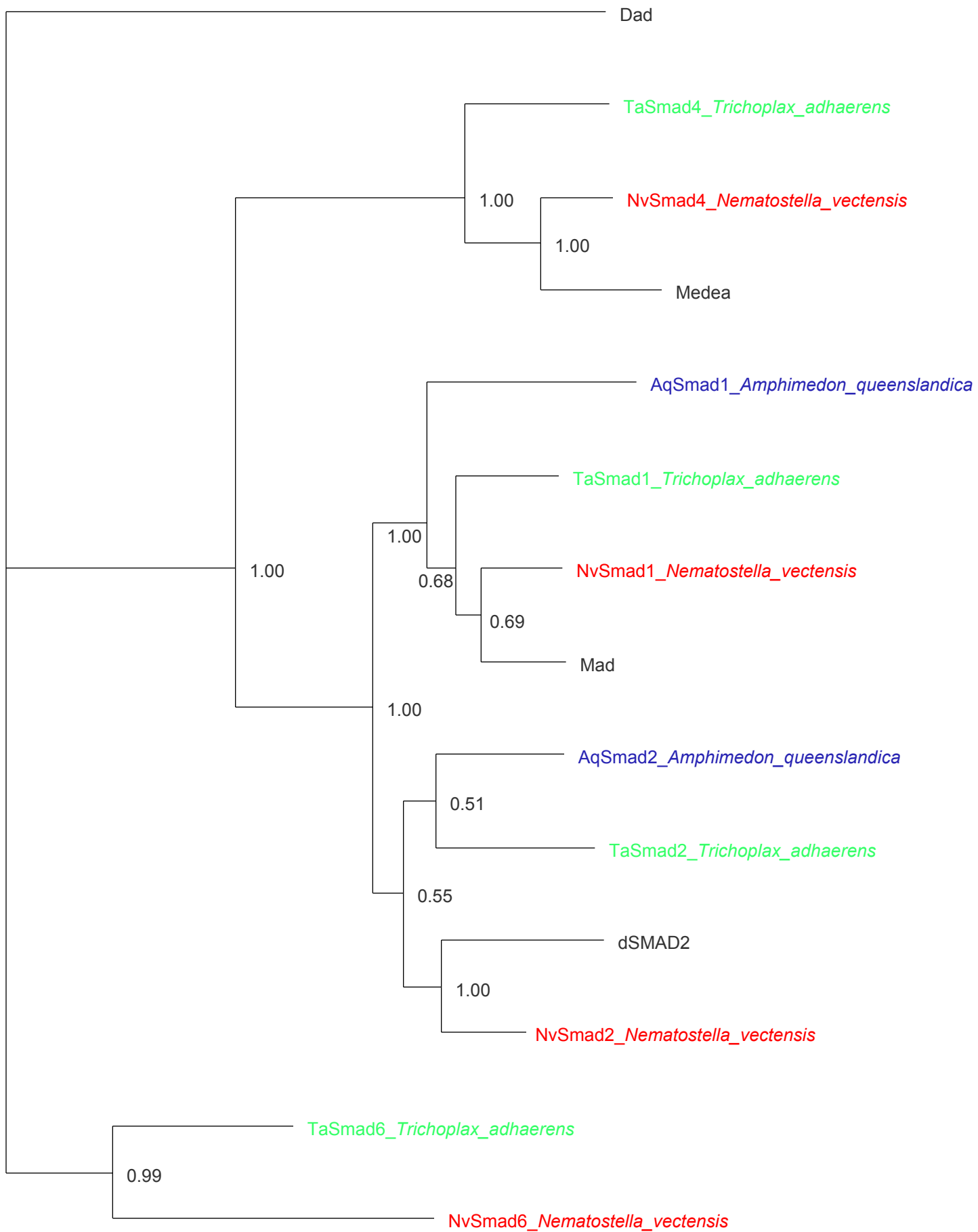

Figure S4

Supplement: Additional file 4 — Figure S4. Two R-Smads (AqSmad1 and AqSmad2) have been detected in Amphimedon genomic traces. The tree is rooted using Dad. [file 1471-2148-9-28-S4.pdf]
