# Supplementary material for: Emergence, development and diversification of the TGF-β signalling pathway within the animal kingdom
Source: BMC Evol Biol. 2009 Feb 3;9:28. doi: 10.1186/1471-2148-9-28 (PMC2657120; doi:10.1186/1471-2148-9-28)
Supplement: Additional file 2 — Figure S2. Receptor phylogenomic tree (rooted on time). View in magnification (at least 200%), in a pdf viewer such as Acrobat Reader, Adobe Acrobat or kpdf. [file 1471-2148-9-28-S2.pdf]

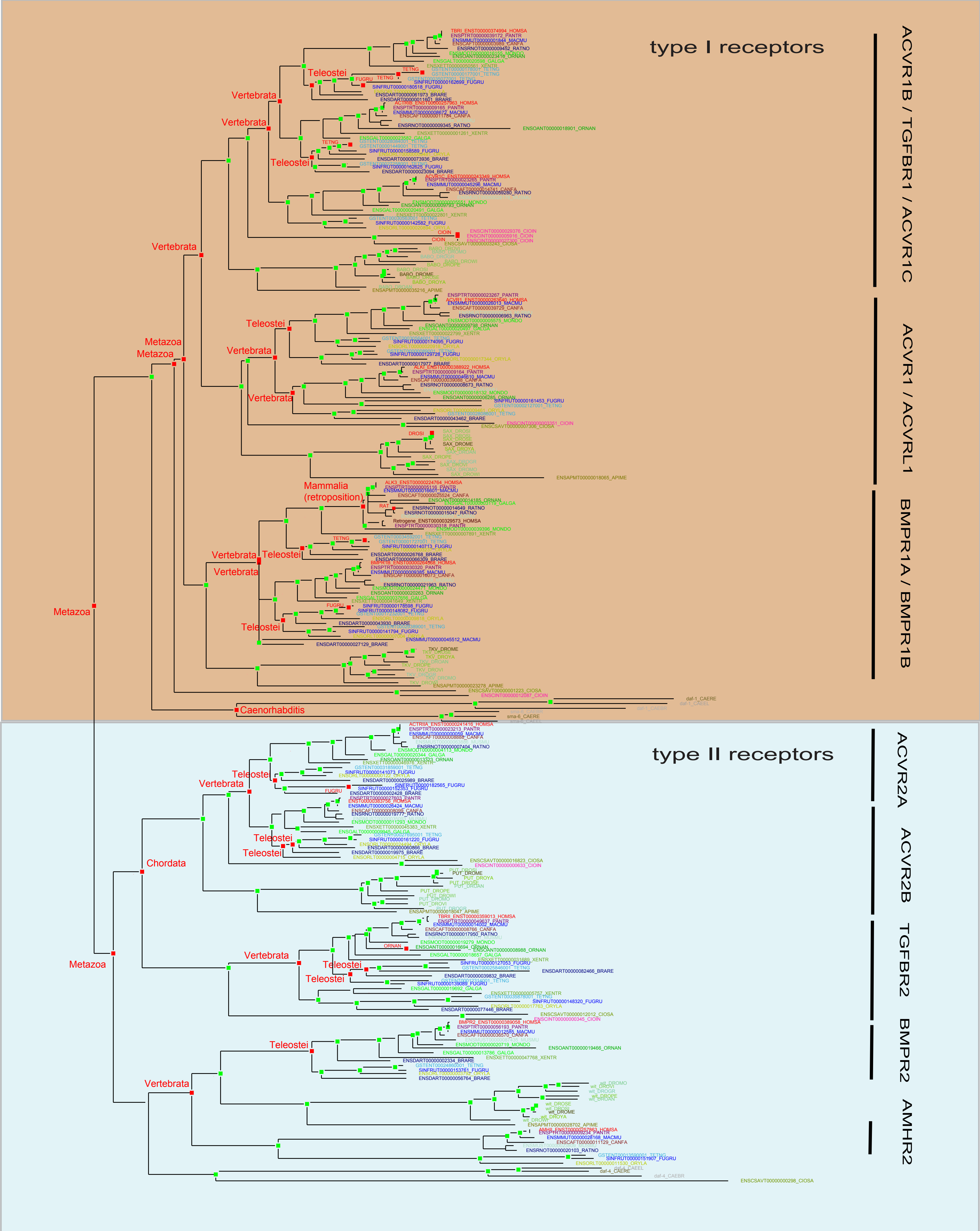

type I receptors
ACVR1B / TGFBR1 / ACVR1C
ACVR1 / ACVRL1
BMPR1A / BMPR1B
Vertebrata
Teleostei
Metazoa
Mammalia (retroposition)
Caenorhabditis
type II receptors
ACVR2A
ACVR2B
TGFBR2
BMPR2
AMHR2
Chordata


Figure S2
